# Advancing Resident Education: Experiential Success in the Creation of a Comprehensive Clinical Didactic Series in Radiation Oncology

**DOI:** 10.1016/j.adro.2024.101452

**Published:** 2024-02-02

**Authors:** Qateeb Khan, Breann Bowar, Bryn Myers, Fatima Kenjar, James D. Byrne, Jordan Gainey

**Affiliations:** a Department of Radiation Oncology, University of Iowa Hospitals and Clinics, Iowa City, Iowa; b Carver College of Medicine, Iowa City, Iowa

## Abstract

**Purpose:**

An effective didactic curriculum is a cornerstone for a successful residency program, as it is the basis upon which residents acquire the necessary knowledge and perspective to provide high-quality, evidence-based care. Here we describe our success in creating a standardized curriculum in clinical radiation oncology – one that was well-received and led to significantly improved performance on the national in-service examination.

**Methods and Materials:**

One-hundred and fifty topics were outlined in accordance with the American Board of Radiology; to accommodate this breadth of material, didactic frequency was increased from biweekly to daily. As a clinical correlate to these sessions, a teaching library of over 100 real-world cases was compiled for individual learning. Finally, comprehensive dosimetric constraints were compiled to aid residents in radiation therapy plan evaluation. To evaluate these curricular changes, anonymous questionnaires were provided to all residents and faculty, and de-identified resident clinical performance from the annual in-service examination was analyzed.

**Results:**

Before the introduction of the standardized curriculum, the mean clinical percentage on the in-service examination was 46%, equivalent to the 17th percentile. Within 2 years of implementation of the new curriculum, both the mean percentage and percentile were significantly improved, with the mean percentage correct at 69.3% and the mean percentile at the 59th percentile (*P* < .001 and *P* = .034, respectively). Feedback showed the curriculum to be well-received and used frequently outside of standard didactic hours.

**Conclusions:**

This is the first report of the creation of a standardized curriculum and outcomes in radiation oncology. Although there are certainly developmental challenges, addressing these barriers creates an education model that effectively imparts knowledge, fosters multidisciplinary thinking, and prepares residents for the diverse challenges of clinical practice. We present our institutional experience with the intent of publishing this curriculum on a national platform in the coming years.

## Introduction

Medical education necessitates a combination of clinical experience and structured didactic instruction. This is widely seen in medical schools, where traditional classroom teaching is complemented by experiential clinical rotations. Unfortunately, the standard medical school curriculum and subsequent preliminary year often do not provide basic training for the radiation oncology resident.^1^^,^^2^ The Accreditation Council for Graduate Medical Education recognizes this need as they make specific mention for structured core didactics among radiation oncology programs.^3^ Furthermore, with the field of oncology undergoing rapid advancements, there is an overt need in radiation oncology for formal, up-to-date learning. Unfortunately, to the detriment of both residents and their departments, the adoption of such structure is widely inconsistent.^4^ There are several factors that play into this challenge. Residency programs are tasked with providing residents both structured teaching and clinical training. This training is often under the apprenticeship model, with clinical demands leading to severe time constraints often at the detriment of didactic time. Although time is certainly one factor that presents an educational challenge, another is the availability, use, and integration of resources. Not only must residency programs financially invest in obtaining pertinent learning materials, they must also provide guidance in their utilization. Faculty are tasked to provide this guidance; yet faculty- specific training in medical education to facilitate learning, provide constructive feedback, and promote reflection is often lacking. These challenges, among others widely present in academic institutions, may lead to deficiencies in resident training. This has gained significant attention recently, with resident board examination performance emerging as a major concern within the radiation oncology community.^5^ Here, we describe our experience in creating a novel comprehensive clinical curriculum, one that was well-received by residents and faculty and demonstrated significantly improved proficiency on standardized examinations.

## Methods and Materials

Historically, didactics at our academic institution were organized as biweekly journal clubs tailored to the month's specific disease site. Landmark trials were selected by faculty, sent to residents, and discussed in an hour-long afternoon session. Resident participation during these sessions was minimal and the overall learning experience was far from comprehensive.

Therefore, to improve on our institutional didactics, the Kern's 6 step approach for curricular development was employed.^6^ Medical residents were surveyed, and the quality of didactics was unanimously deemed as unsatisfactory. Residents expressed the need for a resource that would aid in both clinical preparation and board examinations. Faculty were consulted and asked to provide input about the didactic structure at their previous institutions. After this problem identification and needs assessment, goals and objectives were then outlined.

The American Board of Radiology (ABR) Study Guide for clinical boards served as the basis for outlining the structure of the didactic aspect of the new standardized curriculum.^7^ Topics were created and divided into the following disease sites: benign, gastrointestinal, gynecologic, prostate, genitourinary (nonprostate), hematologic, head and neck, skin, breast, central nervous system, palliation, pediatrics, lung and mediastinum, soft tissue and bone, and biostatistics. For each disease site, content for the didactic lectures was curated from a variety of resources; the highest-yield resources included the National Comprehensive Cancer Network (NCCN) guidelines, Essentials of Clinical Radiation Oncology, Gunderson and Tepper's Clinical Radiation Oncology, eContour, and RadOncQuestions.^8^, ^9^, ^10^, ^11^, ^12^ Example lectures for a disease site are shown in Table 1. In total, 150 PowerPoint based lectures were created. To accommodate this breadth of material necessitated by the ABR, the logistics of didactics were restructured. Rather than biweekly afternoon sessions, didactics were expanded to daily hour-long sessions occurring 5 times a week. Attendance was mandatory and all residents were excused from clinical responsibilities during these sessions. A sample week of didactics is presented in Table 2.

**Table 1:** Sample disease site breakdown

Lung and mediastinal tumors
Anatomy of the lung and mediastinum
Early stage non-small cell lung cancers
Advanced non-small cell lung cancers
The role of postoperative radiation therapy in lung cancer
Recent advances in non-small cell lung cancer
Limited stage small cell lung cancer
Extensive stage small cell lung cancer
Thymomas and other mediastinal tumors
Mesothelioma and pleural tumors
Guest lecture: Systemic therapies in lung cancer
Guest lecture: PFTs, biopsies, and surgeries for thoracic tumors
Journal club 1*
Journal club 2*
Written board prep: Practice questions†
Mock oral boards: Lung and mediastinum

*Abbreviations:* PFT = pulmonary function tests.

⁎ Journal clubs were variable and changed year to year. For example, in the year 2022, journal clubs included Radiation Therapy Oncology Group 0617 and the recent publication of the Lung ART trial.

† Written board prep was conducted on a weekly basis.

**Table 2:** Didactic schedule for week 1 of thoracic malignancies

Day	Monday	Tuesday	Wednesday	Thursday	Friday
Responsible physician	Senior resident	Attending MD (thoracic)	Senior resident	Attending MD (thoracic)	Senior resident
Topic	Foundational knowledge: Early stage NSCLC	Case presentation with RT plan review: SBRT – peripheral, central, and ultracentral tumors	Foundational knowledge: Advanced NSCLC	Case presentation with RT plan review: Conventional ChemoRT	TXIT review: Thorax - NSCLC
Time	1 hour	1 hour	1 hour	1 hour	1 hour

*Abbreviations:* NSCLC = non-small cell lung cancers; RT = radiation therapy; SBRT = stereotactic body RT; TXIT = radiation oncology in-training examination.

Standardization of the daily objectives and guidelines was established in the following structure: Monday and Wednesday sessions emphasized foundational knowledge and were led by senior residents. Tuesday and Thursday sessions were faculty led, translating the previous day's foundational knowledge to real-world patient cases. Faculty sessions were structured in a mock-oral format, using Socratic teaching methods to highlight pearls of contouring, plan evaluation, multidisciplinary tumor board questions, landmark clinical trials, and common insurance prior authorization situations. Finally, Friday sessions were an evaluation session with board style questions done collectively as a resident group; these questions were obtained from both RadOncQuestions and retired in-service examination questions.

The structured daily didactic sessions and content were the cornerstone to the curricular overhaul. However, self-directed learning is undoubtedly essential to medical learning as well.^13^ Although the didactic library was placed in a shared drive for all residents to individually access, 2 additional curricular elements were created to empower residents to take control of their individual learning processes. First, a teaching library of over 100 real-world cases was compiled. This library included initial consult notes, radiation plans, and follow-up notes. Consult notes would be available to understand the workup, clinical decision making, and next steps when a patient first presented to clinic. Imported radiation plans were made available to understand contours and treatment planning, both in a standardized fashion as well as to learn institutional preferences. Finally, follow-up notes were available to recognize long-term toxicities of oncologic treatment as well as to appreciate strategies in oncologic recurrence. A sample case library is presented in Table E1. The second aspect of the curriculum designed to promote individual learning was the compilation of disease-site specific dosimetric constraints to aid in resident plan evaluation. The ABR Study Guide was again referenced for the division of disease sites. Within disease sites, common national and institutional fractionation schedules (ie, stereotactic radiosurgery, stereotactic body radiation therapy, hypofractionation, and conventional fractionation) provided an additional division within the library. As for the dosimetric constraints themselves, resources included NCCN guidelines, American Society for Radiation Oncology (ASTRO) guidelines, American Brachytherapy Society guidelines, HYTEC papers, and landmark clinical trials from the NRG and Radiation Therapy Oncology Group. Sample dose constraints for a disease site are shown in Table E2.

At our institution, the resident cohort is comprised of 7 residents. To objectively evaluate performance, de-identified resident analytics were collected from the annual American College of Radiology radiation oncology in-training examination (ACR TXIT) to determine the efficacy of the curriculum in nationwide standardized testing. The ACR TXIT is widely used among radiation oncology residency programs and offers both programs and residents the opportunity to evaluate their knowledge and identify areas of deficiency relative to their peers.^14^ In addition to this written examination, mock oral examinations were held at a bimonthly frequency scheduled with disease site rotations to allow both the resident and faculty to understand advancement in resident competencies as they completed a rotation.

As for curricular feedback, anonymous questionnaires were provided to all residents and faculty via Qualtrics. These were considered to be of particular value, as in the era of competence-based education standardized examination performance is not adequately reflective of curricular and educational goals. All 7 residents and 9 faculty members were provided surveys. Qualitative and quantitative data were obtained. Questions were designed to assess satisfaction with all aspects of the curriculum. Faculty were surveyed for their opinion about the previous didactic experience as well as the revamped format and content. They were also asked about their perspective on how this transformation affected resident performance in terms of having resources for written and oral examinations, resident clinical readiness, and oncologic competencies. Residents were issued a similar comprehensive survey and individual perspectives were collected regarding the previous didactic structure, new structure, and their board examination and clinical readiness.

### Statistical analysis

For the purposes of this manuscript, the year before the initiation of standardized didactics is termed “T-1.” The year of partial implementation of the curriculum is termed as “pilot year.” This was the year where curricular materials were being created. The first 2 years of the full adoption of the standardized didactic curriculum are termed as “year 1” and “year 2.”

The primary endpoint measured was the mean clinical percentile of all residents as reported on the ACR TXIT. The secondary endpoint measured was the mean clinical percentage correct of all residents as reported on the ACR TXIT. At our institution, the resident cohort was comprised of 7 residents. Of note, in the years 2022 and 2023, the ACR sent updated scores as the initial reports were not accurate. These updated reports, with corrected scores, were used for the purposes of this manuscript.

Comparisons of means across the 4 years were done using a 1-way analysis of variance (ANOVA) with the F-test used to confirm significant differences between years. Levene's statistic was used to confirm the assumption of equal variances. Tukey's post hoc tests were used if the F-test confirmed significant differences between years. Alpha <0.05 was used as the standard to establish statistical significance. All statistical calculations were performed using SPSS version 29.0 (IBM Corp).

This study was approved by the institutional review board as an exempt educational activity.

## Results

**Figure 1:**
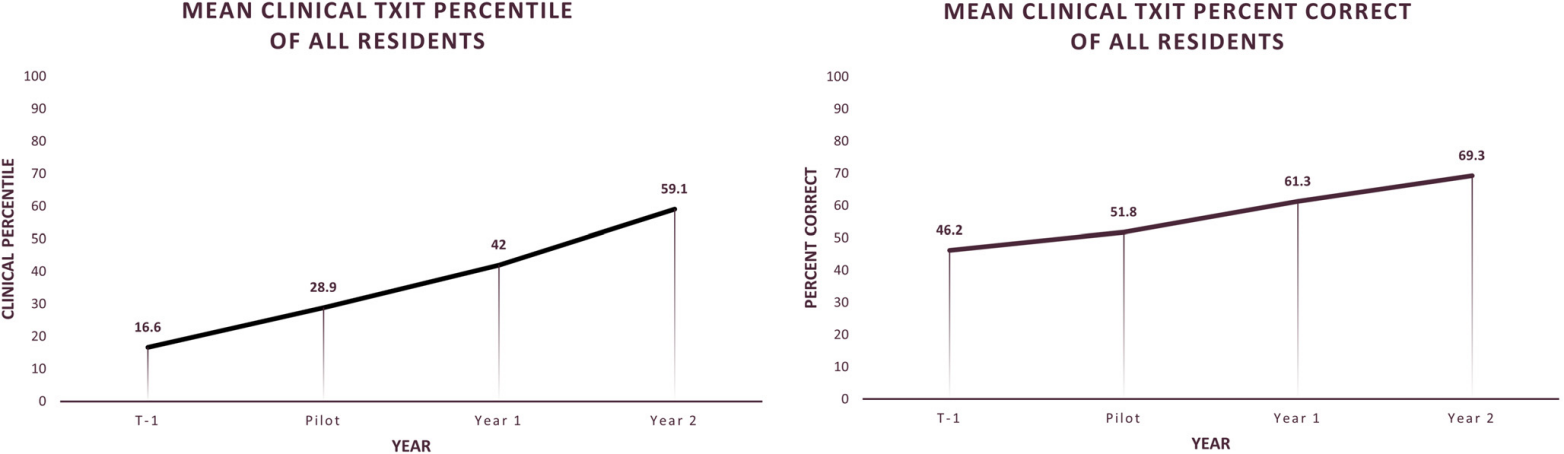
(A) Mean resident clinical percentile on the annual radiation oncology in-training exam charted by year. (B) Mean resident clinical percentage correct on the annual radiation oncology in-training exam charted by year.

The primary endpoint of mean clinical percentile of all residents on the ACR TXIT is presented in Table 3. In the T-1 year, before the introduction of the standardized curriculum, the average resident clinical TXIT score was equivalent to the 17th percentile nationally (±14.1 SD). During the pilot year, this mean percentile increased to the 29th percentile (±25.9 SD). Year 1, the first year of full implementation of the new curriculum, saw an increase in mean percentile to the 42nd percentile (±30.1 SD). By year 2, the mean rose to the 59th percentile (±31.2 SD). This data are represented graphically in Fig. 1A.

**Table 3:** Mean resident clinical percentile and mean clinical percentage correct on the ACR TXIT examination by year, with SD

Year	Mean percentile (SD)	Mean percentage correct (SD)
T-1	16.6th (14.1)	46.2% (6.1)
Pilot	28.9th (25.9)	51.8% (6.4)
Year 1	42.0th (30.1)	61.3% (6.6)
Year 2	59.1st (31.2)	69.3% (12.5)

*Abbreviation:* ACR TXIT = American College of Radiology radiation oncology in-training examination.

A 1-way ANOVA test revealed the improvement in clinical percentile between years to be statistically significant (F = 3.387, between group df = 3, within group df = 24, *P* = .034). Tukey's post hoc tests for the primary endpoint are presented in Table 4 and confirmed that the year 2 percentile was statistically higher than the T-1 percentile, with a mean percentile difference of 42.6 (95% CI, 3.9-81.2; *P* = .027).

**Table 4:** Results of the post hoc test examining differences between years in terms of clinical percentile on the ACR TXIT examination

(I) Year	(J) Year	Mean difference	95% CI		*P* value
		(I-J)	Lower bound	Upper bound	
T-1	Pilot	−12.3	−51.0	26.4	.817
	Year 1	−25.4	−64.1	13.2	.292
	Year 2	−42.6	−81.2	−3.9	.027*
Pilot	T-1	12.3	−26.4	51.0	.817
	Year 1	−13.1	−51.8	25.5	.785
	Year 2	−30.3	−69.0	8.4	.163
Year 1	T-1	25.4	−13.2	64.1	.292
	Pilot	13.1	−25.5	51.8	.785
	Year 2	−17.1	−55.8	21.5	.619
Year 2	T-1	42.6	3.9	81.2	.027*
	Pilot	30.3	−8.4	69.0	.163
	Year 1	17.1	−21.5	55.8	.619

*Abbreviation:* ACR TXIT = American College of Radiology radiation oncology in-training examination.

⁎ Statistically significant result. By convention, “I” refers to the year examined by Tukey's post hoc test, and “J” refers to the year being used as the comparator.

The secondary endpoint of mean clinical percentage correct on the ACR TXIT is presented in Table 3. In the T-1 year, before implementation of the new curriculum, residents across all classes achieved an average of 46.2% of all clinical questions correct (±6.1 SD). In the pilot year, this increased to 51.8% correct (±6.4 SD). By year 1, the mean percentage correct had increased to 61.3% (±6.6 SD), and this continued into year 2 with a mean percentage correct of 69.3% (±12.5 SD). These data are graphically represented in Fig. 1B.

A 1-way ANOVA test revealed that the differences in clinical percent correct between the years were also statistically significant (F = 10.496, between group df = 3, within group df = 24, *P* < .001). Tukey's post hoc tests for the secondary endpoint are presented in Table 5 and had multiple statistically significant differences between years. Year 2 had statistically significantly higher scores than both T-1 (mean difference, 23.1 percentage points; 95% CI, 10.8-35.3; *P* < .001) and the pilot year (mean difference, 17.5 percentage points; 95% CI, 5.2-29.7; *P* = .003). Year 1 was also statistically improved compared with the T-1 year by a mean difference of 15.1 percentage points (95% CI, 2.8-27.3; *P* = .012).

**Table 5:** Results of the post hoc test examining differences between years in terms of clinical percent correct on the ACR TXIT examination

(I) Year	(J) Year	Mean difference	95% CI		*P* value
		(I-J)	Lower bound	Upper bound	
T-1	Pilot	−5.6	−17.9	6.7	.597
	Year 1	−15.1	−27.3	−2.8	.012*
	Year 2	−23.1	−35.3	−10.8	<.001*
Pilot	T-1	5.6	−6.7	17.9	.597
	Year 1	−9.5	−21.7	2.8	.174
	Year 2	−17.5	−29.7	−5.2	.003*
Year 1	T-1	15.1	2.8	27.3	.012*
	Pilot	9.5	−2.8	21.7	.174
	Year 2	−8.0	−20.3	4.3	.299
Year 2	T-1	23.1	10.8	35.3	<.001*
	Pilot	17.5	5.2	29.7	.003*
	Year 1	8.0	−4.3	20.3	.299

*Abbreviation:* ACR TXIT = American College of Radiology radiation oncology in-training examination.

⁎ Statistically significant result. By convention, “I” refers to the year examined by Tukey's post hoc test, and “J” refers to the year being used as the comparator.

**Figure 2:**
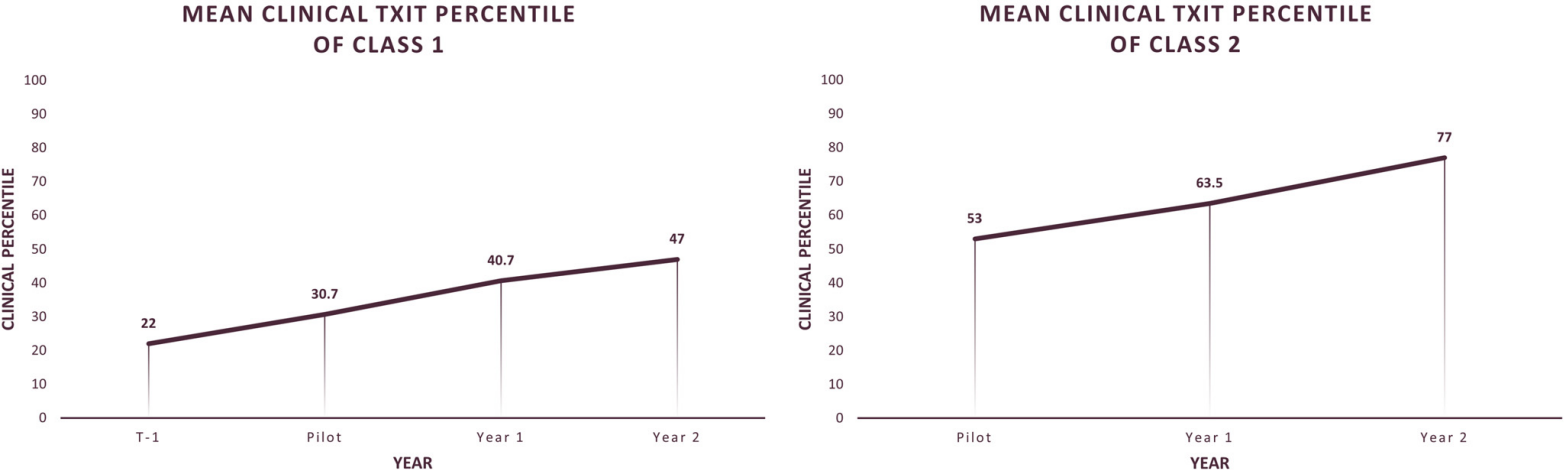
(A) Mean clinical percentile on the annual radiation oncology in-training exam charted by year for resident class 1. (B) Mean clinical percentile on the annual radiation oncology in-training exam charted by year for resident class 2.

Percentile data were also analyzed by resident year. Each resident class taught under the new curriculum demonstrated improved annual clinical scores on the ACR TXIT. Two resident classes were identified as ones that were instructed using the previous curriculum, as part of the T-1 year, who also underwent 2 years of complete implementation of the standardized curriculum, as part of year 1 and 2 cohorts. ACR TXIT clinical performance of these classes, classes 1 and 2, is charted in Fig. 2A and B. Residents in these classes out-paced peers nationally as their clinical percentiles steadily increased year after year.

In addition to demonstrating significant efficacy in improving resident competence, the standardized curriculum was well received among both faculty and residents. Before the introduction of the new curriculum, no resident or faculty member rated the didactic experience as excellent. With the new structure, this was reversed as 86% of faculty and 100% of residents rated the standardized curriculum as excellent. There was unanimous agreement among both faculty and residents that the new curriculum improved residents’ ability to prepare for board examinations and prepare for clinic. Faculty also noted the curriculum improved the residents’ ability to access pertinent literature and guidelines, discuss primary literature, and perform on mock oral examinations. Response rates were 86% among residents and 78% among faculty, and the results are shown in Tables 6 and 7.

**Table 6:** Radiation oncology faculty survey responses regarding the standardized curriculum

Faculty survey questions	Percentage (%)	Count (n)
How would you rate the didactic experience before the current standardized format?		
- Excellent	0%	0
- Good	14%	1
- Average	43%	3
- Poor	29%	2
- Terrible	0%	0
- N/A (not present)	14%	1
How would you rate the current standardized didactic format?		
- Excellent	86%	6
- Good	14%	1
- Average	0%	0
- Poor	0%	0
- Terrible	0%	0
How would you rate the current curricular content?		
- Excellent	100%	7
- Good	0%	0
- Average	0%	0
- Poor	0%	0
- Terrible	0%	0
As a result of the new curriculum, residents have better resources to prepare for the in-service examination.		
- Strongly agree	100%	7
- Agree	0%	0
- Neutral	0%	0
- Disagree	0%	0
- Strongly disagree	0%	0
As a result of the new curriculum, residents have better resources to prepare for the ABR written boards.		
- Strongly agree	100%	7
- Agree	0%	0
- Neutral	0%	0
- Disagree	0%	0
- Strongly disagree	0%	0
As a result of the new curriculum, residents have better resources to prepare for mock oral examinations.		
- Strongly agree	86%	6
- Agree	14%	1
- Neutral	0%	0
- Disagree	0%	0
- Strongly disagree	0%	0
As a result of the new curriculum, residents have better resources to prepare for the ABR oral examination.		
- Strongly agree	86%	6
- Agree	14%	1
- Neutral	0%	0
- Disagree	0%	0
- Strongly disagree	0%	0
As a result of the new curriculum, residents have better resources to prepare for new patient consults.		
- Strongly agree	86%	6
- Agree	14%	1
- Neutral	0%	0
- Disagree	0%	0
- Strongly disagree	0%	0
As a result of the new curriculum, residents have better resources to study oncologic literature.		
- Strongly agree	100%	7
- Agree	0%	0
- Neutral	0%	0
- Disagree	0%	0
- Strongly disagree	0%	0
As a result of the new curriculum, residents have better resources to access national guidelines.		
- Strongly agree	86%	6
- Agree	14%	1
- Neutral	0%	0
- Disagree	0%	0
- Strongly disagree	0%	0
As a result of the new curriculum, residents have better resources to contour and evaluate RT plans with dosimetry.		
- Strongly agree	71%	5
- Agree	29%	2
- Neutral	0%	0
- Disagree	0%	0
- Strongly disagree	0%	0
Narrative feedback:	57%	4

The revised curriculum has greatly improved the residents’ readiness in clinic, case conference, and mock oral examinations. Additionally, it serves as a valuable resource for faculty to efficiently access national guidelines, pivotal trials, and the latest developments in radiation oncology.

With the new curriculum format, the residents have more dedicated time to prepare for key papers and board questions. During the physician-led conference time, the residents are well prepared, and the physician can focus on the clinic cases. The residents then have more hand- on experience for clinic scenarios.

The new standardized curriculum dramatically improves the residents' education. We can easily see the effect of this on patient discussions, notes, and radiation plans.

Thanks for all of your work!

*Abbreviations:* ABR = American Board of Radiology; RT = radiation therapy.

**Table 7:** Radiation oncology resident survey responses regarding the standardized curriculum

Resident survey questions	Percentage (%)	Count (n)
How would you rate the didactic experience before the current standardized format?		
- Excellent	0%	0
- Good	0%	0
- Average	17%	1
- Poor	67%	4
- Terrible	17%	1
- N/A (not present)	0%	0
How would you rate the current standardized didactic format?		
- Excellent	100%	6
- Good	0%	0
- Average	0%	0
- Poor	0%	0
- Terrible	0%	0
How often do you use the content of the standardized curriculum?		
- Daily	50%	3
- 4-6 times a week	17%	1
- 2-3 times a week	33%	2
- Once a week	0%	0
- Once a month	0%	0
As a result of the new curriculum, I have better resources to prepare for the in-service examination.		
- Strongly agree	100%	6
- Agree	0%	0
- Neutral	0%	0
- Disagree	0%	0
- Strongly disagree	0%	0
As a result of the new curriculum, I have better resources to prepare for the ABR clinical boards.		
- Strongly agree	100%	6
- Agree	0%	0
- Neutral	0%	0
- Disagree	0%	0
- Strongly disagree	0%	0
As a result of the new curriculum, I have better resources to prepare for mock orals.		
- Strongly agree	83%	5
- Agree	17%	1
- Neutral	0%	0
- Disagree	0%	0
- Strongly disagree	0%	0
As a result of the new curriculum, I have better resources to prepare for the ABR oral boards.		
- Strongly agree	83%	5
- Agree	17%	1
- Neutral	0%	0
- Disagree	0%	0
- Strongly disagree	0%	0
As a result of the new curriculum, I have better resources to discuss oncology literature with attendings.		
- Strongly agree	100%	6
- Agree	0%	0
- Neutral	0%	0
- Disagree	0%	0
- Strongly disagree	0%	0
As a result of the new curriculum, I have better resources to access national guidelines.		
- Strongly agree	100%	6
- Agree	0%	0
- Neutral	0%	0
- Disagree	0%	0
- Strongly disagree	0%	0
As a result of the new curriculum, I have better resources to discuss RT plan cases with dosimetry.		
- Strongly agree	100%	6
- Agree	0%	0
- Neutral	0%	0
- Disagree	0%	0
- Strongly disagree	0%	0
Narrative feedback:	100%	6

The standardized curriculum helped consolidate pertinent literature and topics into readily available and organized material for assistance with completing resident tasks and objectives. Excellent study resource for clinic and boards.

The new curriculum was well-rounded and encompassed all the necessary educational topics in radiation oncology. Because of this my learning was more efficient. Rather than having to look something up in multiple different resources before finding the information I needed, I was able to use the created PowerPoints as a summary of all the resources. If I needed to then go more in-depth, I could find what resource was used for that information and go directly to it. I was able to prepare for simple patient consults quickly and focus my time on learning contouring and preparing for complex patients and rare disease sites. I was also introduced to contouring atlases and guidelines early via the curriculum, which benefitted learning the correct contouring from an early time in residency.

Since the new change, this residency has been more focused on clinical learning. It is helpful to have current and well thought-out PowerPoints to refer to while studying and preparing for clinic. Our current curriculum is an excellent resource for self-study outside of didactic sessions. I use these materials for my board studying. It is nice to have a resource to refer back to frequently to ensure I am using evidence-based guidelines for patients of all disease sites. This curriculum allows us to do this efficiently as everything is expertly organized.

The curriculum has significantly improved my educational experience. I know this implementation will ultimately make me a better doctor and help me to provide patients with better care.

The new curriculum was a major update that significantly improved resident didactics and learning at all radiation oncology resident levels, including faculty learning and participation.

The fundamentals of each disease site are more clear.

*Abbreviations:* ABR = American Board of Radiology; RT = radiation therapy.

## Discussion

Radiation oncology is a distinctive specialty in which readiness for clinical practice hinges on a delicate balance between experiential clinical exposure and structured formal instruction. This learning often begins during Post-Graduate Year-2 year of residency as the Association of American Medical Colleges does not mandate clinical oncologic experience in medical school, creating a fundamental lack of exposure to basic oncologic medicine among medical graduates.^15^ In a survey of radiation oncology residents in the early 2000s, around 70% had 2 to 3 months of exposure to radiation oncology before residency and the rest had less.^4^ Although there have been efforts in standardized radiation oncology didactics among medical students, this exposure is often in the fourth year of medical school, almost 2 years from the start of radiation oncology residency.^16^ Furthermore, internship year is collectively viewed as of minimal benefit in learning oncologic medicine, with a minority of residents viewing the year as beneficial in advancing their development as a radiation oncologist.^2^

Therefore, the burden of training the radiation oncology resident lies almost entirely on the resident and their program. Unfortunately, with the extreme variation of didactics among programs, radiation oncology residents are often inadequately prepared to manage patients as they begin residency.^17^ Although there certainly is a plethora of resources, the learning curve in the first few months of residency is steep. Consulting new patients regarding their oncologic care plans, understanding the fundamentals of contouring, and correctly evaluating radiation therapy plans are all skills that require structured education. Being able to locate and navigate high-yield resources, including but not limited to NCCN guidelines, ASTRO Clinical Practice Guidelines, American Brachytherapy Society Guidelines, E-Contour, RadoncQuestions, and the Mednet takes a fair degree of time, introduction, direction, and guidance. Without structured curricula, Post-Graduate Year-2 residents often haphazardly learn the basic principles of radiation oncology, handicapping not only their growth as residents but also the efficiency of the department.^4^ Both program directors and residents have expressed the need to formalize curricula, yet the resources created in this specific effort are both scarce and underdeveloped.^18^ There have been great initiatives by the radiation oncology community, from the American College of Radiation Oncology deck curriculum to the Radiation Oncology Virtual Education Rotation educational panels, yet the creation of a standardized clinical curriculum and its outcomes is work that is warranted and long overdue.^19^^,^^20^ Other specialties have reported success in such curricular efforts, yet none are described in radiation oncology.^21^^,^^22^ Our experience, detailed here, has shown that this effort is feasible, clinically beneficial, and significantly improves resident performance on standardized testing.

Subjectively, our curriculum was well-received among both faculty and residents. Among the faculty, the vast majority rated the new curriculum as having excellent format and content. They expressed how residents had better resources to prepare for written and oral examinations. Faculty also noted how the new curriculum “greatly improved residents’ readiness in clinic.” Residents themselves expressed a similar perspective, as the new curriculum efficiency consolidated study resources and significantly improved the learning experience across all resident levels. Perhaps the greatest reason for this reception was the consistency this curriculum provided. Under the traditional apprenticeship model of residency, individual resident learning is quite variable based on attending clinical case load and volume.^23^ Without a formalized curriculum, there are inherent disparities in structured didactics due to the preferences of incumbent leadership. With our standardization, all residents received a consistent level of didactic teaching, helping them to build a strong foundational knowledge in oncology. There was reduced variability between years in the quality of the didactics, as foundational materials were simply updated from year to year. Moreover, given the modular structure of the curriculum, the latest clinical trial findings and updated guidelines were often presented to residents just a few short days after their national publication. For example, when the Federation of Gynecology and Obstetrics staging for endometrial cancer was updated in 2023, it was presented in a group setting just a few days after publication with an introductory analysis on its effect on clinical decision making. Aside from content creation and presentation, a special focus was placed on the structure and accessibility to curricular content. Every component of the curriculum was efficiently saved and organized, with all content being immediately accessible. From the PowerPoints in a shared resident drive to the radiation therapy case library, there was a clear framework for residents and faculty to access these materials for personalized learning and reference. Residents would routinely access these materials for reference during clinic days, and faculty members would use the curricular content in their practice to stay updated on the latest clinical trials and national guidelines. During teaching sessions with residents outside of the daily didactic sessions, faculty would often adapt these teaching materials, highlighting the comprehensive and multifunctional nature of this curricular effort. Several faculty members also used curricular content in their own presentations to national audiences. Overall, both residents and faculty clearly demonstrated in their feedback that this standardized curriculum elevated the learning experience at our institution, not only to perform well on examinations but to provide high quality patient care in their practices as well.

Objectively, our curricular efforts demonstrated significant efficacy on the national radiation oncology in-service examination. In the past decade, radiation oncology resident performance on board examinations has come under a fair degree of scrutiny. The ABR's mission statement states that “diplomates [must] demonstrate the requisite knowledge, skill, and understanding of their disciplines to the benefit of patients.”^24^^p1^ Within the radiation oncology community, there have been several calls to restructure these examinations to accurately test oncologic competence.^25^^,^^26^ Although this initiative is certainly warranted and has elicited a response from the ABR, there is an equal need for residency programs to adequately prepare residents for these examinations.^27^ Cramming and using vacation time to study for these examinations is a common technique used among residents.^28^ Although possibly effective for test taking, this study style does not accomplish the goal of residency in terms of true understanding and long-term retention.^29^ To address these longstanding educational concerns, there has been a push to create an ASTRO subcommittee of graduate medical education, charged to develop and maintain a comprehensive radiation oncology curriculum.^5^^,^^30^ We achieved this goal at an institutional level, creating a true longitudinal resource that would aid residents throughout residency and beyond. Not only did the entire resident cohort perform better on the in-service examination, each resident class demonstrated marked year-to-year improvement, highlighting the efficacy of this effort for the individual resident.

It must be emphasized that clinical excellence goes far beyond ABR and ACR examinations.^31^ Although these board examinations no doubt play a crucial role in the journey of a radiation oncologist, national educational frameworks are moving toward a model of competency-based education.^32^ At our program, we implemented a system of teaching, assessment, and feedback where residents were placed at the center of their own education and faculty were able to provide targeted feedback and competency assessment to aid the residents in their educational journeys. Our intent was not merely to produce excellent test scores; it was to enable training physicians to provide high-quality oncologic care. With our newly created institutional resources, providing such care was efficiently and confidently accomplished. Strengths of our paper include the subjective reporting of the popularity of this curriculum as well as the objective reporting of resident performance on the national in-service examination. In 2016, MD Anderson Cancer Center reported their experience in developing a comprehensive curriculum, one that was well received by both residents and faculty.^23^ This work by Dr Holiday and colleagues demonstrated the feasibility and importance of creating a comprehensive institutional curriculum. However, no objective standardized testing data were reported. Moreover, our curriculum included various novel components that significantly contributed to resident learning, including the dose constraint library and the radiation therapy case library. To our knowledge, this is the first report of a comprehensive institutional curriculum with marked success in both subjective and national objective evaluations. Inherent limitations present within this project are the single institutional experience of this curriculum and the small sample size of our cohort. However, initial results of the curriculum are promising, providing a model for radiation oncology residency curricula on a national level.

## Conclusion

There is an overt need in radiation oncology residencies for the creation of a comprehensive didactic curriculum. Although there are certainly challenges in its development, from the hundreds of hours of content creation to the coordination of multidisciplinary lectures, it is paramount that residency programs place a special emphasis on residents’ education and their clinical success. We developed a unique didactic curriculum that significantly aided residents in their clinical learning and intend to publish the content on a national platform in the coming years.

## Disclosures

The authors declare that they have no known competing financial interests or personal relationships that could have appeared to influence the work reported in this paper.

## Appendix Supplementary materials

Supplementary Material Files Image, application 1

## Footnotes

Supplementary material associated with this article can be found in the online version at doi:10.1016/j.adro.2024.101452.
