# Supplementary material for: Advancing Resident Education: Experiential Success in the Creation of a Comprehensive Clinical Didactic Series in Radiation Oncology
Source: Adv Radiat Oncol. 2024 Feb 2;9(5):101452. doi: 10.1016/j.adro.2024.101452 (PMC10999511; doi:10.1016/j.adro.2024.101452)
Supplement: Supplementary Material Files [file mmc1.docx]

**Supplementary Tables**

**Supplementary Table 1.** Radiotherapy Case Library for Thoracic Malignancies

| **Thoracic Malignancies Radiotherapy Plans** |
| --- |
| Peripheral SBRT |
| Central SBRT |
| Ultracentral SBRT |
| Advanced Non-Small Cell Lung Cancer |
| Superior Sulcus Tumor |
| Postoperative Radiotherapy in Non-Small Cell Lung Cancer |
| Limited Stage – Small Cell Lung Cancer |
| Extensive Stage – Small Cell Lung Cancer |
| Thymoma |
| Mesothelioma |

**Supplementary Table 2.** Sample dose constraint breakdown. Residents were instructed to always use principles of ALARA (as low as reasonably possible), as for certain dose constraints the allowed radiation dose would be far greater than what would be probable on an individual case (i.e.: V_20Gy_ on a 5 fraction SBRT plan).

| **Thoracic Dose Constraints** | | | | |
| --- | --- | --- | --- | --- |
| - | **3 Fraction SBRT** | **5 Fraction SBRT** | **Hypofractionated** | **Conventionally Fractionation** |
| **Prescription Dose** | **54 Gy in 3 fx** | **50 Gy in 5 fx** | **60 Gy in 15 Fx** | **60 Gy in 30 fx** |
| Brachial Plexus | D_max_ < 24 Gy | D_max_ < 32 Gy | D_max_ < 50 Gy | D_max_ < 66-70 Gy  Median < 69 Gy |
| Spinal Cord | D_max_ < 18 Gy | D_max_ < 30 Gy | D_max_ < 36 Gy | D_max_ < 50 Gy  D_max_ < 41 Gy (for BID SCLC) |
| Proximal Bronchial Tree | D_max_ < 30 Gy | D_max_ < 52.5 Gy | D_max_ < 66 Gy | D_max_ < 69 Gy |
| Heart / Pericardium | D_max_ < 30 Gy | D_max_ < 52.5 Gy | D_max_ < 66 Gy | V_50Gy_ < 25%  Mean < 20 Gy |
| Great Vessels | D_max_ < 45 Gy | D_max_ < 52.5 Gy | D_max_ < 66 Gy | D_max_: 76 Gy |
| Esophagus | D_max_ < 27 Gy | D_max_ < 52.5 Gy | D_max_ < 48 Gy | V_60Gy_ < 17%  Mean < 34 Gy |
| Lungs | V_20Gy_ < 10-15%  Mean < 8 Gy | V_20Gy_ < 10-15%  Mean < 8 Gy | V_18Gy_ <37%  Mean < 18 Gy | V_20Gy_ < 35%  Mean < 20 Gy |
| Rib | V_30Gy_ < 30-70 cc  D_max_ < 30 Gy | V_30Gy_ < 30-70 cc  D_max_ < 57 Gy | D_max_ < 66 Gy | D_max_ < 66 Gy |
| Skin | D_max_ < 24 Gy | D_max_ < 32 Gy | D_max_ < 45 Gy | D_max_ < 45 Gy |
